# Acupuncture decreased the risk of coronary heart disease in patients with rheumatoid arthritis in Taiwan: a Nationwide propensity score-matched study

**DOI:** 10.1186/s12906-018-2384-5

**Published:** 2018-12-22

**Authors:** Mei-Yao Wu, Ming-Cheng Huang, Hou-Hsun Liao, Jen-Huai Chiang, Yu-Chen Lee, Chung-Y Hsu, Mao-Feng Sun, Hung-Rong Yen

**Affiliations:** 1 Department of Chinese Medicine, China Medical University Hospital, 2 Yude Road, North District, Taichung, 404 Taiwan; 2 Graduate Institute of Chinese Medicine, School of Chinese Medicine, College of Chinese Medicine, China Medical University, 91 Hsueh-Shih Road, North District, Taichung, 404 Taiwan; 3 Dalin Tzu Chi Hospital, Buddhist Tzu Chi Medical Foundation, Chiayi, 622 Taiwan; 4 Management Office for Health Data, China Medical University Hospital, Taichung, 404 Taiwan; 5 School of Medicine, College of Medicine, China Medical University, Taichung, 404 Taiwan; 6 Graduate Institute of Acupuncture Science, College of Chinese Medicine, China Medical University, Taichung, 404 Taiwan; 7 Graduate Institute of Clinical Medical Science, China Medical University, Taichung, 404 Taiwan; 8 Research Center for Traditional Chinese Medicine, Department of Medical Research, China Medical University Hospital, 2 Yude Road, North District, Taichung, 404 Taiwan; 9 Research Center for Chinese Herbal Medicine, China Medical University, Taichung, 404 Taiwan; 10 Chinese Medicine Research Center, China Medical University, Taichung, 404 Taiwan; 11 Department of Biotechnology, Asia University, Taichung, 413 Taiwan

**Keywords:** Acupuncture, Coronary heart disease, National Health Insurance Research Database, Rheumatoid arthritis, Taiwan

## Abstract

**Background:**

Patients with rheumatoid arthritis (RA) have a higher risk of coronary heart disease (CHD). Acupuncture, a commonly used treatment for patients with RA, has not been reported to prevent CHD in patients with RA. We aimed to assess the risk of developing CHD in acupuncture users and non-users of patients with RA.

**Methods:**

We identified 29,741 patients with newly diagnosed RA from January 1997 to December 2010 from the Registry of Catastrophic Illness Patients Database from the Taiwanese National Health Insurance Research Database. Among them, 10,199 patients received acupuncture (acupuncture users), and 19,542 patients did not receive acupuncture (no-acupuncture users). After performing 1:1 propensity score matching by sex, age, baseline comorbidity, conventional treatment, initial diagnostic year, and index year, there were 9932 patients in both the acupuncture and no-acupuncture cohorts. The main outcome was the diagnosis of CHD in patients with RA in the acupuncture and no-acupuncture cohorts.

**Results:**

Acupuncture users had a lower incidence of CHD than non-users (adjusted HR = 0.60, 95% CI = 0.55–0.65). The estimated cumulative incidence of CHD was significantly lower in the acupuncture cohort (log-rank test, *p < .001*). Subgroup analysis showed that patients receiving manual acupuncture of traditional Chinese medicine style, electroacupuncture, or combination of both all had a lower incidence of CHD than patients never receiving acupuncture treatment. The beneficial effect of acupuncture on preventing CHD was independent of age, sex, diabetes mellitus, hypertension, hyperlipidemia, and statins use.

**Conclusions:**

This is the first large-scale study to reveal that acupuncture might have beneficial effect on reducing the risk of CHD in patients with RA. This study may provide useful information for clinical utilization and future studies.

## Background

Rheumatoid arthritis (RA) is one of the most prevalent chronic and systemic inflammatory disorders, with the characteristics of joint destruction, functional disability, and early death [1]. Autoantibody production, synovial inflammation, cartilage and bone destruction are the main pathological findings in RA patients [2]. Current pharmacotherapies of RA include non-steroidal anti-inflammatory drugs (NSAIDs), glucocorticoids, and disease-modifying anti-rheumatic drugs (DMARDs) [3]. DMARDs, including synthetic and biological DMARDs, have been demonstrated to suppress inflammation to reduce structural damage.

The high mortality rate of patients with RA was mainly because of the significantly higher risk of cardiovascular diseases in patients with RA than in the general population [4]. The previous study revealed that patients with RA had an approximately twofold higher risk of coronary heart disease (CHD) [5]. The risk of CHD in patients with RA is highly associated with the traditional risk factors of CHD, which include hypertension, diabetes mellitus (DM), hyperlipidemia, smoking, obesity, and physical inactivity [6]. Chronic inflammation and immune dysregulation play an important role in inducing atherosclerosis and endothelial dysfunction in patients with RA [7]. Among current pharmacotherapy of RA, methotrexate (MTX) might prevent ischemic cardiovascular disorders in patients with RA [8]. Etanercept, a tumor necrosis factor-α (TNF-α) inhibitor, was demonstrated to attenuate endothelial dysfunction in a rat model [9]. These findings suggest that anti-inflammatory therapy may reduce the CHD risk in patients with RA.

A previous cohort study revealed that approximately 27.3% of patients with RA were traditional Chinese medicine (TCM) users in Taiwan, and approximately 23.6% of TCM users with RA received acupuncture [10]. The anti-inflammatory mechanisms of acupuncture have been demonstrated in murine models [11, 12]. Previous clinical trials have demonstrated that acupuncture suppressed inflammation in patients with RA [13, 14]. However, no prior study evaluated whether acupuncture can prevent CHD in patients with RA. To investigate whether acupuncture decreases the incidence of CHD in patients with RA, we conducted this cohort study.

## Methods

### Data sources

The Taiwanese National Health Insurance Research Database (NHIRD) is a good database for the cohort study because it contains the long-term follow-up data of most of the population in Taiwan. The National Health Insurance (NHI) program was implemented in 1995 in Taiwan by the National Health Insurance Administration. TCM services have been reimbursed through the NHI since 1996 [15]. The reimbursed population reached more than 99.5% in 2015 [16]. NHIRD is a big database that can prevent sampling bias [17].

The data source of this study was the Registry of Catastrophic Illness Patients Database; patients with autoimmune diseases, such as RA [18], systemic lupus erythematosus, multiple sclerosis, Sjögren syndrome, and cancer, are included in this database, which is a subsection of the NHIRD [19]. All of the patients with RA in the database are given catastrophic illness certificates based on the clinical and laboratory diagnoses by rheumatologists. The NHI program waived RA patients’ copayments for receipt of RA-related treatments, including Western medicine and TCM. In this database, the International Classification of Diseases, Ninth Revision, Clinical Modification (ICD-9-CM) codes were the most commonly used diagnostic codes. Because the NHIRD contains identified secondary data for research, the present study was waived from informed consent. This study was approved by the Research Ethics Committee of China Medical University and Hospital, Taichung, Taiwan (CMUH104-REC2–115).

### Study cohort identification and covariates

**Fig. 1:**
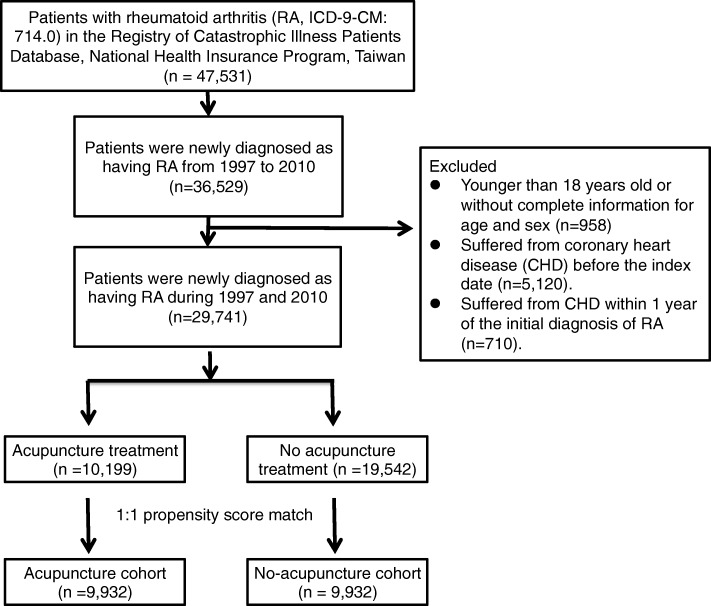
Study population flowchart diagram. Using the inclusion and exclusion criteria, we identified 10,199 patients who underwent acupuncture and 19,542 patients who never underwent acupuncture. After performing 1:1 propensity score matching, there were 9932 patients in the acupuncture and no-acupuncture cohorts

Patients who were newly diagnosed with RA (ICD-9-CM code 714.0) between January 1, 1997 and December 31, 2010 were identified, as shown in Fig. 1. Patients younger than 18 years old or with incomplete information on the age and sex were excluded. Patients who received acupuncture between the initial diagnosis of RA and happening of CHD or December 31, 2010 were identified as the acupuncture cohort. In the acupuncture cohort, we defined the index date as the first time that the patients started to receive acupuncture. The immortal time of the acupuncture cohort was the period between the initial diagnosis of RA and the index date. Patients who suffered from CHD were identified through ICD-9-CM codes 410, 411, 413, 414.0, 414.8, and 414.9. Patients diagnosed as having CHD before the index date or within one year after their initial diagnosis of RA were excluded. Patients were followed up until December 31, 2011 or withdrawn from the NHIRD.

To reduce confounding factors, we used 1:1 propensity-score matching by sex, age (per 5 years), comorbidity, conventional drug use, the initial diagnostic year of RA, and the index year through multiple logistic regression analysis. The comorbidities analyzed in our study included DM (ICD-9-CM: 250), hypertension (ICD-9-CM: 401–405), hyperlipidemia (ICD-9-CM: 272), congestive heart failure (ICD-9-CM: 402.01, 402.11, 402.91, 404.01, 404.03, 404.11, 404.13, 404.91, 404.93, and 428.0), cerebral vascular diseases (ICD-9-CM: 430–438), depression (ICD-9-CM: 296.2–296.3, 300.4, and 311), anxiety (ICD-9-CM: 300.0, 300.2, 300.3, 308.3, and 308.91), alcoholism (ICD-9-CM: 291, 303, 305.00–305.03, 790.3, and V11.3), tobacco dependence (ICD-9-CM: 305.1), and obesity (ICD-9-CM: 278 and A183). Patients with comorbidity were identified if the ICD-9-CM codes of the selected comorbidity appeared two or more times in the outpatient or inpatient claims. The conventional drugs analyzed in our study included MTX, hydroxychloroquine, sulfasalazine, TNF-α inhibitors, oral steroids, NSAIDs, and statins. Finally, equal numbers of patients in the acupuncture and no-acupuncture cohorts were analyzed in this study. Patients were divided into 3 groups according to their age: 18–39 years old, 40–59 years old, and older than 60 years old.

### Types of acupuncture and disease categories in the acupuncture cohort

**Fig. 2:**
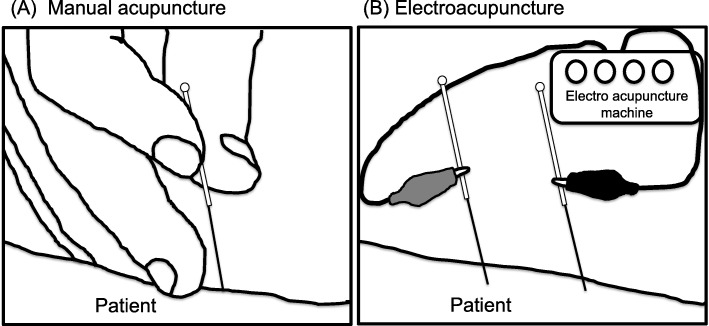
Diagram of the two types of acupuncture treatment. **a** Manual acupuncture of traditional Chinese medicine type (**b**) Electroacupuncture

We analyzed the acupuncture type that patients received by the treatment codes, which included manual acupuncture of TCM type (B41, B42, B45, B46, B80-B84, B90-B94, P27041, P31103, P32103, and P33031) and electroacupuncture (B43, B44, B86–89, and P33032). Figure 2 illustrated these two types of acupuncture treatments.

### Statistical analyses

Statistical analysis was performed using SAS 9.4 (SAS Institute, Cary, NC, U.S.A.), and *p* < 0.05 in two-tailed tests indicate statistical significance. The difference of baseline characteristics between the acupuncture and no-acupuncture cohorts was expressed as standardized mean differences. Standardized mean differences with less than 0.2 s.d. indicated a negligible difference in the mean or proportions between the two cohorts. For each variable, we used Cox proportional hazard regression to analyze the hazard ratios (HRs) and 95% confidence interval (95% CI). The Kaplan-Meier method was used to determine the cumulative incidence of CHD in both cohorts, and the log-rank test was used to compare incidence curves between the acupuncture and no-acupuncture cohorts.

## Results

The identified patient numbers in both cohorts were equal after performing the 1:1 propensity score matched method (Fig. 1). There were no significant differences in the sex, age, baseline comorbidity, and conventional drug use between the no-acupuncture and acupuncture cohorts (Table 1). The included patients were female predominant with a mean age at approximately 50 years old in both cohorts. Almost all patients in both cohorts have used NSAIDs, and more than 90% of patients have also used oral steroids. Approximately 15% of patients took statins in both the no-acupuncture and acupuncture cohorts. Manual acupuncture of the TCM type was the most commonly used acupuncture treatment.

**Table 1:** Characteristics of rheumatoid arthritis patients according to whether they received acupuncture treatment

Variable	Rheumatoid arthritis Underwent acupuncture				Standardized mean difference
	No (*n* = 9932)		Yes (*n* = 9932)		
	n	%	n	%	
Sex					
Female	8212	82.68	8225	82.81	0.003
Male	1720	17.32	1707	17.19	0.003
Age group					
18–39	2267	22.83	2013	20.27	0.003
40–59	6099	61.41	6659	67.05	0.003
≥60	1566	15.77	1260	12.69	0.003
Mean ± SD (years)	50.39 (13.7)		50.36 (12.53)		0.002
Baseline Comorbidity					
Diabetes mellitus	1211	12.19	1193	12.01	0.006
Hypertension	2358	23.74	2339	23.55	0.005
Hyperlipidemia	1488	14.98	1475	14.85	0.004
Congestive heart failure	94	0.95	107	1.08	0.013
Stroke	741	7.46	731	7.36	0.004
Depression	631	6.35	618	6.22	0.005
Anxiety	1525	15.35	1499	15.09	0.007
Alcoholism	20	0.20	24	0.24	0.009
Tobacco dependence	27	0.27	30	0.30	0.006
Obesity	80	0.81	77	0.78	0.003
Drug use					
Methotrexate	7131	71.80	7130	71.79	0
Hydroxychloroquine	8372	84.29	8423	84.81	0.014
Sulfasalazine	7289	73.39	7278	73.28	0.003
TNF-α inhibitors	1137	11.45	1151	11.59	0.004
NSAIDs	9908	99.76	9909	99.77	0.002
Oral steroids	9449	95.14	9438	95.03	0.005
Statins	1449	14.59	1438	14.48	0.003
Types of acupuncture					
Manual acupuncture of TCM type			8614	86.73	
Electroacupuncture			340	3.42	
Combination of manual acupuncture and electroacupuncture			978	9.85	
Immortal time, days (mean, median)	1064.07 (989.63)		1044.96 (1056.50)		
Acupuncture visits, mean			9.81		
Follow-up time, years (mean, median)	4.02 (3.03)		4.91 (3.44)		

During the follow-up period, 909 patients in the acupuncture cohort and 1233 patients in the no-acupuncture cohort developed CHD (Table 2). Compared with the no-acupuncture cohort, the incidence of CHD was significantly lower in the acupuncture cohort (adjusted HR = 0.60, 95% CI = 0.55–0.65). RA patients with old age, DM, or hypertension were prone to developing CHD.

**Table 2:** Cox model with hazard ratios and 95% confidence intervals for coronary heart disease in patients with rheumatoid arthritis who did or did not receive acupuncture treatment

Variable	No. of event (*n* = 2142)	Crude^a^			Adjusted^b^		
		HR	(95%CI)	*p*-value	HR	(95%CI)	*p*-value
Acupuncture							
No	1233	1.00	reference		1.00	reference	
Yes	909	0.63	(0.58–0.69)	<.0001	0.60	(0.55–0.65)	<.0001
Sex							
Female	1692	1.00	reference		1.00	reference	
Male	440	1.32	(1.19–1.47)	<.0001	1.21	(1.09–1.34)	0.0005
Age group							
18–39	126	1.00	reference		1.00	reference	
40–59	1458	4.22	(3.52–5.07)	<.0001	3.85	(3.20–4.63)	<.0001
≥60	548	8.76	(7.22–10.64)	<.0001	5.35	(4.37–6.56)	<.0001
Baseline Comorbidity (ref = non-site comorbidity)							
Diabetes mellitus	443	2.25	(2.03–2.50)	<.0001	1.46	(1.30–1.64)	<.0001
Hypertension	877	2.65	(2.43–2.89)	<.0001	1.77	(1.61–1.95)	<.0001
Hyperlipidemia	391	1.64	(1.47–1.83)	<.0001	1.08	(0.95–1.22)	0.2342
Congestive heart failure	33	1.94	(1.37–2.73)	0.0002	1.00	(0.71–1.42)	0.9783
Cerebral vascular diseases	267	2.09	(1.84–2.38)	<.0001	1.22	(1.07–1.40)	0.0037
Depression	127	1.18	(0.99–1.42)	0.0664	0.97	(0.80–1.17)	0.7509
Anxiety	312	1.20	(1.07–1.36)	0.0028	0.94	(0.83–1.07)	0.3402
Alcoholism	2	0.54	(0.14–2.17)	0.3886	0.51	(0.13–2.05)	0.3427
Tobacco dependence	1	0.25	(0.04–1.74)	0.1606	0.22	(0.03–1.57)	0.1316
Obesity	18	1.38	(0.87–2.20)	0.171	1.15	(0.72–1.83)	0.5623
Drug use							
Methotrexate	1210	0.47	(0.43–0.51)	<.0001	0.64	(0.58–0.70)	<.0001
Hydroxychloroquine	1680	0.60	(0.55–0.67)	<.0001	0.80	(0.720–.89)	<.0001
Sulfasalazine	1437	0.66	(0.61–0.73)	<.0001	0.91	(0.82–1.00)	0.0559
TNF-α inhibitors	105	0.35	(0.29–0.43)	<.0001	0.48	(0.39–0.59)	<.0001
NSAIDs	2130	1.36	(0.34–5.44)	0.6638	1.73	(0.43–6.93)	0.4412
Oral steroids	2010	0.66	(0.55–0.79)	<.0001	0.82	(0.68–0.98)	0.033
Statins	350	1.07	(0.96–1.20)	0.2408	0.69	(0.61–0.78)	<.0001

Crude HR^a^ represents the relative hazard ratio. Adjusted HR^b^ represents the adjusted hazard ratio mutually adjusted for acupuncture use, age, sex, diabetes mellitus, hypertension, hyperlipidemia, congestive heart failure, cerebral vascular diseases, depression, anxiety, alcoholism, tobacco dependence, obesity, Methotrexate, Hydroxychloroquine, Sulfasalazine, TNF-α inhibitors, oral steroids, NSAIDs and statins in Cox proportional hazard regression

**Fig. 3:**
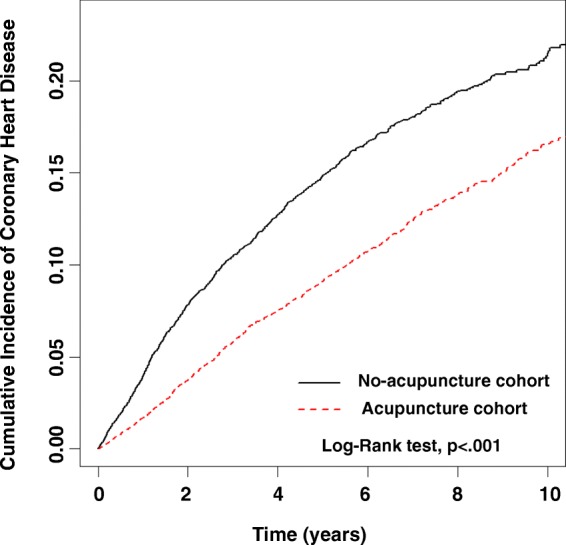
Cumulative incidence of coronary heart disease (CHD) between the acupuncture and no-acupuncture cohorts. The cumulative incidence of coronary heart disease in the acupuncture cohort (dashed line) is significantly lower than the no-acupuncture cohort (solid line) (log-rank test, *p* < .001). The zero point indicated the index date

The beneficial effect of acupuncture on the incidence of CHD was observed in both women and men (Table 3, adjusted HR = 0.62 in women, 95% CI = 0.56–0.68; adjusted HR = 0.58 in men, 95% CI = 0.48–0.70). Acupuncture significantly decreased the incidence of CHD in all age groups. Regardless of whether patients had DM, hypertension, hyperlipidemia, or stroke, acupuncture lowered the risk of CHD. Moreover, the beneficial effect of acupuncture in preventing CHD was independent of conventional drug use, including statins, MTX, hydroxychloroquine, sulfasalazine, oral steroids, and TNF-α inhibitors. Overall, the cumulative incidence of CHD from the index date was significantly lower in the acupuncture cohort (Fig. 3).

**Table 3:** Incidence rates, hazard ratio and confidence intervals for coronary heart disease in rheumatoid arthritis patients who did or did not receive acupuncture treatment, stratified by sex, age, comorbidity and drug use

Variables	Rheumatoid arthritis						Compared with no-acupuncture cohort	
	No acupuncture (*n* = 9932)			Acupuncture (*n* = 9932)			Crude HR (95%CI)	Adjusted HR (95%CI)
	Event	Person-years	IR	Event	Person-years	IR		
Total	1223	39,930.25	30.63	909	48,742.08	18.65	0.63(0.58–0.69)***	0.60(0.55–0.65)***
Sex								
Female	970	33,542.81	28.92	722	40,610.75	17.78	0.64(0.58–0.7)***	0.62(0.56–0.68)***
Male	253	6387.44	39.61	187	8131.32	23.00	0.60(0.50–0.73)***	0.58(0.48–0.70)***
Age group								
18–39	81	10,544.53	7.68	45	10,665.24	4.22	0.56(0.39–0.81)**	0.53(0.37–0.77)***
40–59	816	24,725.99	33.00	642	32,628.29	19.68	0.61(0.55–0.68)***	0.59(0.53–0.66)***
≥60	326	4659.72	69.96	222	5448.55	40.74	0.61(0.51–0.72)***	0.62(0.52–0.73)***
Baseline Comorbidity								
Diabetes mellitus								
No	969	36,055.56	26.88	720	43,658.67	16.49	0.64(0.58–0.70)***	0.60(0.55–0.66)***
Yes	254	3874.69	65.55	189	5083.41	37.18	0.58(0.48–0.71)***	0.59(0.49–0.72)***
Hypertension								
No	717	32,065.40	22.36	538	38,485.07	13.98	0.64(0.57–0.72)***	0.62(0.56–0.70)***
Yes	506	7864.85	64.34	371	10,257.00	36.17	0.59(0.51–0.67)***	0.58(0.51–0.67)***
Hyperlipidemia								
No	1000	35,475.99	28.19	741	43,080.05	17.20	0.63(0.58–0.70)***	0.61(0.56–0.67)***
Yes	223	4454.26	50.06	168	5662.03	29.67	0.59(0.49–0.73)***	0.57(0.47–0.70)***
Congestive heart failure								
No	1202	39,641.02	30.32	897	48,354.94	18.55	0.63(0.58–0.69)***	0.61(0.56–0.66)***
Yes	21	289.23	72.61	12	387.14	31.00	0.44(0.22–0.90)*	0.50(0.24–1.07)
Cerebral vascular diseases								
No	1069	37,549.80	28.47	796	45,604.09	17.45	0.64(0.58–0.70)***	0.60(0.55–0.66)***
Yes	154	2380.45	64.69	113	3137.99	36.01	0.58(0.45–0.74)***	0.59(0.46–0.76)***
Depression								
No	1144	38,013.07	30.09	861	46,405.65	18.55	0.64(0.59–0.70)***	0.62(0.56–0.67)***
Yes	79	1917.19	41.21	48	2336.42	20.54	0.51(0.36–0.73)***	0.51(0.35–0.73)***
Anxiety								
No	1042	35,170.64	29.63	778	43,056.56	18.07	0.63(0.58–0.69)***	0.60(0.55–0.66)***
Yes	181	4759.61	38.03	131	5685.51	23.04	0.62(0.50–0.78)***	0.62(0.49–0.78)***
Alcoholism								
No	1222	39,865.91	1222	908	48,660.02	908	0.63(0.58–0.69)***	0.61(0.56–0.66)***
Yes	1	64.34	1	1	82.05	1	0.58(0.03–9.89)	–
Tobacco dependence								
No	1222	39,862.01	1222	909	48,661.38	909	0.63(0.58–0.69)***	0.61(0.56–0.66)***
Yes	1	68.24	1	0	80.70	0	–	–
Obesity								
No	1214	39,697.39	30.58	900	48,463.26	18.57	0.63(0.58–0.69)***	0.61(0.55–0.66)***
Yes	9	232.86	38.65	9	278.81	32.28	0.95(0.37–2.42)	0.74(0.26–2.09)
Drug use								
Methotrexate								
No	530	10,045.02	52.76	392	13,042.58	30.06	0.60(0.53–0.68)***	0.61(0.54–0.7)***
Yes	693	29,885.23	23.19	517	35,699.50	14.48	0.64(0.57–0.72)***	0.60(0.53–0.67)***
Hydroxychloroquine								
No	257	5500.43	46.72	195	6736.19	28.95	0.65(0.54–0.78)***	0.62(0.52–0.75)***
Yes	966	34,429.82	28.06	714	42,005.89	17	0.63(0.57–0.69)***	0.60(0.54–0.66)***
Sulfasalazine								
No	410	9296.59	44.10	285	11,957.00	23.84	0.57(0.49–0.66)***	0.54(0.47–0.63)***
Yes	813	30,633.66	26.54	624	36,785.07	16.96	0.66(0.59–0.73)***	0.64(0.57–0.71)***
TNF-α inhibitors								
No	1157	34,731.45	33.31	870	42,448.62	20.50	0.64(0.59–0.7)***	0.62(0.56–0.67)***
Yes	66	5198.80	12.70	39	6293.45	6.20	0.48(0.32–0.72)***	0.42(0.28–0.62)***
NSAIDs								
No	0	42.45	0	2	62.67	31.91	–	–
Yes	1223	39,887.80	30.66	907	48,679.41	18.63	0.63(0.58–0.69)***	0.61(0.56–0.66)***
Oral steroids								
No	68	1442.40	47.14	54	1799.74	30	0.69(0.48–1.00)*	0.74(0.51–1.07)
Yes	1155	38,487.85	30.01	855	46,942.34	18.21	0.63(0.57–0.69)***	0.60(0.55–0.65)***
Statins								
No	1020	33,516.65	30.43	762	41,308.09	18.45	0.63(0.58–0.69)***	0.61(0.55–0.67)***
Yes	203	6413.60	31.65	147	7433.99	19.77	0.63(0.51–0.78)***	0.59(0.48–0.73)***

Abbreviations: IR, incidence rates per 1000 person-years; HR, hazard ratio; and CI, confidence interval

Adjusted HR: adjusted for acupuncture use, age, sex, diabetes mellitus, hypertension, hyperlipidemia, congestive heart failure, cerebral vascular diseases, depression, anxiety, alcoholism, tobacco dependence, obesity, Methotrexate, Hydroxychloroquine, Sulfasalazine, TNF-α inhibitors, oral steroids, NSAIDs and statins in Cox proportional hazard regression

*: *p* < 0.05; **: *p* < 0.01; and ***: *p* < 0.001

Subgroup analysis was performed to evaluate whether different types of acupuncture had any correlation and difference on preventing CHD in patients with RA (Table 4). Patients receiving only manual acupuncture, only electroacupuncture, or combination of manual acupuncture and electroacupuncture all had a lower incidence of CHD than patients never receiving acupuncture.

**Table 4:** Cox model for coronary heart disease in patients with rheumatoid arthritis receiving different types of acupuncture treatment vs. not receiving acupuncture treatment

	n	CHD (*n* = 2132)	Crude HR^*^(95%CI)	Adjusted HR^†^ (95%CI)
No-acupuncture	9932	1223	1 (reference)	1 (reference)
Acupuncture				
Manual acupuncture of TCM type	8614	819	0.67(0.62–0.74)***	0.64(0.58–0.69)***
Electroacupuncture	340	19	0.48(0.31–0.76)**	0.46(0.29–0.73)**
Combination of manual acupuncture and electroacupuncture	978	71	0.39(0.30–0.49)***	0.36(0.28–0.46)***

Abbreviations: HR, hazard ratio; CHD, coronary heart disease; CI, confidence interval; and TCM, traditional Chinese medicine

Crude HR^*^ represents relative hazard ratio. Adjusted HR^†^ represents adjusted hazard ratio mutually adjusted for age, sex, diabetes mellitus, hypertension, hyperlipidemia, congestive heart failure, cerebral vascular diseases, depression, anxiety, alcoholism, tobacco dependence, obesity, Methotrexate, Hydroxychloroquine, Sulfasalazine, TNF-α inhibitors, oral steroids, NSAIDs and statins in Cox proportional hazard regression

**: *p* < 0.01; and ***: *p* < 0.001

Among the different types of CHD, patients with acupuncture had a significant lower incidence of chronic ischemic heart disease (Table 5). However, there was no significant difference in the incidence of angina pectoris between the two groups. The effect on acute myocardial infarction was not analyzed (data not shown; only 11 patients had acute myocardial infarction in two groups).

**Table 5:** Cox model for angina pectoris and chronic ischemic heart disease in patients with rheumatoid arthritis receiving acupuncture treatment vs. not receiving acupuncture treatment

		N	Angina pectoris			Chronic ischemic heart disease		
			n	Crude HR^*^	Adjusted HR^†^	n	Crude HR^*^ (95%CI)	Adjusted HR^†^ (95%CI)
Acupuncture								
No		8733	24	1 (reference)	1 (reference)	516	1 (reference)	1 (reference)
Yes		9044	21	0.79(0.44–1.43)	0.72(0.4–1.3)	358	0.59(0.52–0.68)***	0.56(0.49 − 0.64)***

Crude HR^*^ represents relative hazard ratio. Adjusted HR^†^ represents adjusted hazard ratio mutually adjusted for age, sex, diabetes mellitus, hypertension, hyperlipidemia, congestive heart failure, cerebral vascular diseases, depression, anxiety, alcoholism, tobacco dependence, obesity, Methotrexate, Hydroxychloroquine, Sulfasalazine, TNF-α inhibitors, oral steroids, NSAIDs and statins in Cox proportional hazard regression

***: *p* < 0.001

Table 6 showed the top five disease categories, which represented the reasons for clinical visits, in the acupuncture cohort. Most patients with RA received acupuncture because of musculoskeletal disease.

**Table 6:** The top five disease categories (as the reasons for clinical visits) in acupuncture user cohort

Disease (ICD-9-CM)	Acupuncture users (*n* = 9932)	
	n	%
Musculoskeletal system and connective tissue (710–739)	6885	69.32
Injury (800–999)	5377	54.14
Symptoms, signs and ill-defined conditions (780–799)	700	7.05
Nervous system (320–389)	463	4.66
Digestive system (520–579)	302	3.04

Maximal of three ICD-9-CM codes could be recorded in one visit

## Discussion

To the best of our knowledge, this nationwide population-based study is the first report to reveal that acupuncture decreased the risk of CHD in patients with RA. Our study found that the beneficial effects of acupuncture for developing CHD in patients with RA were independent of sex, age, DM, and hypertension. Moreover, acupuncture decreased the risk of CHD in patients with RA who took statins, which was reported to reduce the risk of cardiovascular diseases [20]. Among different types of CHD, our results demonstrated that patients with acupuncture had a significant lower incidence of chronic ischemic heart disease than patients without receiving acupuncture treatment.

The risk of CHD in patients with RA is highly associated with the conventional risk factors of CHD, such as hypertension, DM, hyperlipidemia, and physical inactivity [6]. Our study also demonstrated that RA patients with comorbidities, including DM, hypertension, or hyperlipidemia, had a higher risk of CHD. Even in RA patients with DM, hypertension, or hyperlipidemia, our study indicated that acupuncture decreased the incidence of CHD. Previous clinical trials indicated that acupuncture improved swelling and range of motion of knee joints, physical activity, and quality of life in patient with RA [14, 21, 22]. Improved physical activity might be one of the possible explanations that acupuncture can prevent CHD in patients with RA. However, we also found that for those RA patients with the comorbidity of congestive heart failure, acupuncture did not reduce the risk of CHD.

The previous study stated that endothelial dysfunction contributed to atherosclerosis and induced cardiovascular disorders [23]. The current conventional drugs for RA treatment, including glucocorticoids [24] and etanercept (a TNF-α inhibitor) [9], have been demonstrated to improve endothelial dysfunction through the anti-inflammatory effect in RA rat models. However, the protective effect of glucocorticoids remains controversial because glucocorticoids also enhance the risk of cardiovascular disorders based on their negative effects on blood pressure, lipid, insulin resistance and obesity [25]. The protective effect of MTX at a low dose against CHD in patients with RA was also demonstrated in a previous meta-analysis study although it did not attenuate hyperlipidemia or insulin resistance [8]. Our study also indicated that patients with RA who took MTX or TNF-α inhibitors had a lower risk of CHD. In addition, regardless of whether patients used TNF-α inhibitors or MTX, acupuncture significantly decreased the risk of CHD in those patients.

Chronic inflammation plays an important role in the pathogenesis of RA and its comorbidities, including DM, hypertension, and CHD [7]. As a result, amelioration of inflammatory activity should be considered in the CHD preventive strategy for patients with RA. The anti-inflammatory mechanisms of acupuncture have been evaluated in murine models of inflammatory disorders [11, 12]. Acupuncture corrected the imbalance between Th17 and Treg cells to attenuate the severity of RA in a rat RA model [26]. IL-17, cytokine secreted by Th17 cells, has been demonstrated to be important in the pathogenesis of RA, and it has been demonstrated to be associated with CHD [27]. Further mechanistic studies are needed to evaluate whether acupuncture can suppress IL-17 in RA to prevent CHD. Previous clinical trials have demonstrated that acupuncture suppressed inflammation in patients with RA [13, 14]. Based on these studies, acupuncture not only had a traditionally recognized analgesic effect, it had an anti-inflammatory effect. Anti-inflammation might be one of the mechanisms by which acupuncture decreased the incidence of CHD in RA patients. In addition, previous studies demonstrated that the combination of acupuncture with current conventional treatment had additive effects on hypertensive patients [28] and diabetic patients [29], which could contribute to how acupuncture decreased the risk of CHD in RA patients with comorbidity or conventional treatment. The mechanistic study is needed in the future. We have established a collagen-induced arthritis murine model and designed a clinical trial to investigate whether acupuncture is effective on the immunological modulation in RA.

The strength of this study was the comprehensive large-scale database, Taiwan’s NHIRD, which provides a large sample size and reduces selection bias while including long-term follow-up data [17]. Although there were a few clinical trials investigating the efficacy of acupuncture for the treatment of patients with RA [13, 14, 21, 22, 30–35], none of them addressed the question whether acupuncture treatment reduces the risk of cardiovascular disease among patients with RA.

Some limitations merit attention in our study. First, the detailed information about severity of RA, including pain scores, the severity of inflammation, or the destruction of cartilage and bone were not provided in the database. Because patients with different severities may receive different conventional treatments, we performed a 1:1 propensity score matching to minimize this confounding factor. We found that there were no difference in percentages of patients who used analgesic drugs, including NSAIDs and oral steroids, in the acupuncture and no-acupuncture cohorts. The second limitation is that lifestyle data, such as smoking, alcohol drinking, and body mass index (BMI), were unavailable in the datasets. We were able to acquire information of the illnesses that resulted from these personal habits and lifestyles, including alcoholism, tobacco dependence, and obesity. However, an underestimation of smoking exposure and alcohol drinking in both cohorts still may exist. The third limitation was that the datasets did not provide detailed information of selected acupoints and treatment protocols. Usually, TCM doctors selected acupoints for each patient according to the TCM diagnosis, which may vary during the disease progression. In addition, the duration or visit times of acupuncture in the current study might be underestimated because the NHI program only covers maximal 15 times of acupuncture per month and maximal 2–3 times per week, some patients may use self-pay for additional acupuncture treatment, which could not be identified from the NHIRD database. Future clinical trials should consider comparing the effects of a set of fixed acupoints versus flexible acupoints and different duration/times of acupuncture treatment. Although there are some limitations in the current retrospective cohort study, it’s a “real-world evidence” that can reflect actual use in practice [36] from a comprehensive large-scale database. Evidences gathered from future clinical trials should be able to provide more information regarding the selection of acupoints.

## Conclusions

Our study revealed that acupuncture treatment might have beneficial effect on reducing the risk of CHD in patients with RA in Taiwan. This noteworthy finding can provide useful information for further clinical and mechanistic studies.

## Abbreviations

95% CI: 95% confidence interval
CHD: Coronary heart disease
DM: Diabetes mellitus
DMARDs: Disease-modifying anti-rheumatic drugs
HRs: Hazard ratios
ICD-9-CM: International Classification of Diseases, Ninth Revision, Clinical Modification
MTX: Methotrexate
NHI: National Health Insurance
NHIRD: National Health Insurance Research Database
NSAIDs: Non-steroid anti-inflammatory drugs
RA: Rheumatoid arthritis
TCM: Traditional Chinese medicine
